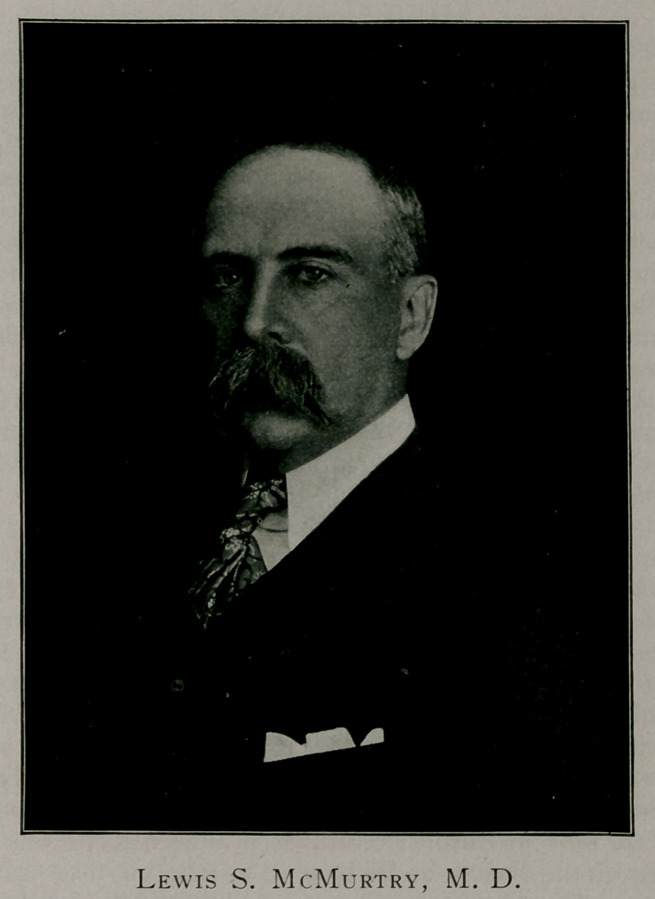# The American Medical Association at Atlantic City

**Published:** 1904-07

**Authors:** 


**A Monthly Review of Medicine and Surgery.**

*EDITOR:*

WILLIAM WARREN POTTER, M. D.

All communications, whether of a literary or business nature, books for review and exchanges should be addressed to the editor: 284 FRANKLIN STREET, BUFFALO, N. Y.

VOL. XLIII.—LIX. JULY, 1904. No. 12

### The American Medical Association at Atlantic City.

THE fifty-fifth annual meeting of the American Medical Association presents some distinguishing features that deserve consideration. The experiment of holding two meetings at one place within the short period of four years was tried at Atlantic City, and proved successful in a marked degree. The registration of 2,890 members and visitors was unprecedented in the history of the association, being nearly 900 more than the total registration at the same place in 1900.

One reason for the increase in these figures is the nearly accomplished unification of the profession in the state of New York. Recognising this fact, the President of the American Medical Association courteously invited the members of the Medical Society of the State of New York to attend and participate in the scientific and social features of the meeting. It appears that about 250 members availed themselves of this privilege, which indicates that the rank and file of the profession in this state recognise the fact that the schism which has existed for twenty-two years is at an end, the formal action of the supreme court only being required to officially terminate it.

Another element contributing toward success is the improved organisation of the national body, which manifested itself strongly at Atlantic City. Following the method of the Medical Society of the State of New York, which for nearly a century has made the county society the basic unit of representation, the American Medical Association a few years ago adopted a similar plan, and its advantages became apparent in the increased attendance and smooth working of affairs at the last meeting. The house of delegates, a new feature inaugurated at Saratoga two years ago, also showed increased strength and great improvement as a legislative body.

The scientific work of the association was never better done than at this meeting. The addresses on medicine, surgery and state medicine, one and all, were of a high order, while the section work kept pace with the spirit of progress that everywhere prevails in scientific bodies. The president's address, too, is to

LEWIS S. MCMURTRY, M. D.

be reckoned as a state paper of dignity and importance. All these conditions conspired to make the meeting of 1904 memorable, and one that may be called the best in its history without disparagement to any that have preceded it.

The election of officers resulted as follows: president, Lewis S. McMurtry, Louisville, Ky.; vice-presidents, Edward Jackson, Denver, Colo.; James Hall Bell, San Antonio, Texas; F. C. Shattuck, Boston, Mass.; B. C. Pennington, Atlantic City, N. J.; secretary, George H. Simmons, Chicago; treasurer, Frank Billings, Chicago; trustees, T. J. Happel, Trenton, Tenn.; W. W. Grant, Denver, Colo.; Philip Marvel, Atlantic City, N. J.; place of meeting, Portland, Ore.; orators,—address in medicine, Charles G. Stockton, Buffalo, N. Y.; address in surgery, John Collins Warren, Boston, Mass.; address in state medicine, George Blumer, San Francisco, Cal.

### President McMurtry.

Dr. Lewis S. McMurtry, of Louisville, the president-elect, is a representative of the best type of the American physician of the present period. Born at Harrodsburg, Ky., September 14, 1850, his early life was developed in that rich agricultural region, famous alike for its blue grass and the strength of character of its citizens. His Scotch ancestry bespeaks a sturdiness of manhood which is typified in the sweet life of his mother, now more than ninety years old and still in the enjoyment of clear faculties and splendid physical health. His early education was obtained at Center College, Danville, from which he received the baccalaureate degree in 1870, which was supplemented by the degree of M. A., in 1874. He received his doctorate degree from Tulane University, New Orleans, in 1873, and immediately entered the Charity Hospital in that city, where he served a year as interne and chief of clinic.

Dr. McMurtry began the practice of his profession at Danville in 1874. In 1881, he became professor of anatomy in the Kentucky School of Medicine, at Louisville. He devoted a portion of 1883 to the study of gynecologic and pelvic surgery at Philadelphia. While residing at Danville he was appointed chairman of the McDowell memorial committee, by the Kentucky State Medical Society, and through his active work the memorial was completed and the monument dedicated May 14, 1879.

Dr. McMurtry was elected president of the Kentucky State Medical Society in 1888. He spent a portion of the winter and spring of 1889 in Europe, returning in season to preside at the meeting of the society at Richmond, in May of that year. He became professor of gynecology and abdominal surgery in the Hospital College of Medicine at Louisville in 1890, and soon afterward was elected president of the faculty, this college being the medical department of Central University. He became one of the founders of the American Association of Obstetricians and Gynecologists in 1888, and served as its president in 1893. He was also a founder of the Southern Surgical and Gynecological Association, of which he was president in 1891. In 1883, he served as a member of the first board of trustees of the American

Medical Association and played an active part in establishing the *Journal* of that body on an enduring basis. Dr. McMurtry's Infirmary at Louisville is concededly as fine a private hospital in its location, equipment and life-saving record as has ever been established in this country. His record as a surgeon in successful work performed in the abdominal and pelvic cavities is not second to that of any man, living or dead. He is a hard worker, a brilliant operator, and an indefatigable attendant at the bedside until his patient is safely convalescent.

Dr. McMurtry has contributed liberally to the literature of medicine, especially as relates to gynecology and abdominal surgery. His papers cover a wide field of subjects and are found in society transactions and journals published during the last twenty-five years. He also contributed the chapter on surgery of the uterus to the International Textbook of Surgery, as well as several chapters to Reed's Textbook of Gynecology. He is gynecologist to Saints Mary and Elizabeth hospital, to the Gray Infirmary and to the Louisville city hospital. He is a Fellow of the Edinburgh Obstetrical Society, of the British Gynecological Society, a corresponding member of the Philadelphia Obstetrical Society, and an honorary member of the Medical Society of the State of New York. He was one of the founders of the International Periodical Congress of Gynecology and Obstetrics, and of the Pan-American Medical Congress, acting as president of the section on obstetrics and gynecology at the first meeting of the latter at Washington, in 1893.

From the foregoing it will be observed that Dr. McMurtry has been an active member of the American Medical Association for more than twenty years, during which time he has served as chairman of the section on obstetrics and gynecology, as chairman of several important committees, as a member of the board of trustees, and of the house of delegates. He is, therefore, thoroughly conversant with all the details of the organisation from the lowest step to the highest, and his selection as president is the direct outgrowth of a long series of duties well performed and may well be regarded as a reward for faithful, meritorious, and eminent service rendered the association and the profession of medicine.

## Figures and Tables

**Figure f1:**